# Gitelman-like syndrome after cisplatin therapy: a case report and literature review

**DOI:** 10.1186/1471-2369-7-10

**Published:** 2006-05-24

**Authors:** Kessarin Panichpisal, Freddy Angulo-Pernett, Sharmila Selhi, Kenneth M Nugent

**Affiliations:** 1 Department of Internal Medicine, Texas Tech University Health Sciences Center, 3601 4^th ^Street, Lubbock, Texas, 79430-79410, USA

## Abstract

**Background:**

Cisplatin is a well-known nephrotoxic antineoplastic drug. Chronic hypokalemic metabolic alkalosis with hypomagnesemia and hypocalciuria is one of the rare complications associated with its use.

**Case presentation:**

A 42- year-old woman presented with a 20 year-history of hypokalemic metabolic alkalosis with hypomagnesemia and hypocalciuria after cisplatin-based chemotherapy for ovarian cancer. This patient has had chronic muscle aches and fatigue and has had episodic seizure-like activity and periodic paralysis. Only thirteen other patients with similar electrolyte abnormalities have been described in the literature. This case has the longest follow-up.

**Conclusion:**

Cisplatin can cause permanent nephrotoxicity, including Gitelman-like syndrome. This drug should be considered among the possible causes of chronic unexplained electrolyte disorders.

## Background

Cisplatin is the most commonly used antineoplastic agent for the treatment of solid tumors and is a well-known cause of nephrotoxicity [1]. In this study we report a case with chronic hypokalemic metabolic alkalosis with hypomagnesemia and hypocalciuria 20 years after the initial treatment with cisplatin therapy. Only three literature reports have described the association of these electrolyte abnormalities and cisplatin therapy; our case has the longest follow-up. This entity is phenotypically identical to the autosomal recessive renal tubular disorder described by Gitelman et al [2].

## Case presentation

This 42-year-old woman was diagnosed with ovarian cancer in 1986 when she was 23 years old. She had undergone a physical examination and laboratory tests during the prior year with normal results. (These normal laboratory results are no longer available.) She was treated with surgery and 12 courses of cisplatin, adriamycin, and cyclophosphamide. Several months after finishing her chemotherapy, she developed right-sided facial and arm twitching (possible seizures suggested by a neurologist), carpopedal spasm, and paresthesias in extremities. Head CT and EEG were normal. Laboratory tests showed hypokalemia (2.5 mEq/l), hypocalcemia (7.8 mg/dl), and hypomagnesemia (0.2 mg/dl). From1986 to 2003, she noted muscle aches and fatigue. Her plasma K^+ ^levels ranged from 1.2 to 7.2 mEq/l (n = 44 measurements; 23 values = 3.5 mEq/L), and her Mg^++ ^level ranged from 0.2 to 2.4 mg/dl. (n = 44 measurements; 43 values < 2.1 mEq/L). She required daily treatment with oral KCL 20–80 mEq, Mg oxide 200–2,400 mg, amiloride 5–20 mg, and spironolactone 100–200 mg to maintain her electrolytes in or near the normal range. She was admitted for IV potassium administration one time in 2002.

In April and August 2003, she had two episodes of hypokalemic paralysis; plasma K^+ ^levels were 1.2 and 1.6 mEq/l despite taking oral KCL 80 mEq daily. She was then placed on IV potassium 20 mEq and oral KCL 160 mEq daily. In August 2003, her Mg^++ ^level was 1.0 mg/dl on oral Mg oxide 4000 mg daily, and she was started on IV magnesium sulfate 4 grams weekly.

In January 2004 she presented to our clinic to establish a new primary care physician. She had muscle aches, diarrhea, paresthesias, and depression. She denied vomiting and diuretic use. She has had diabetes mellitus for 2 years. There was no family history of any electrolyte disorders. Her medications included magnesium sulfate 4 grams IV weekly, and daily oral KCL 240 mEq, amiloride 5 mg, aldactone 150 mg and avandia 8 mg. Her height was 158 cm; weight 74.1 kg; BMI 29.6; blood pressure 100/80 mm Hg; and pulse 80/min. Her physical examination was unremarkable. Laboratory data are listed in Table 1. Her renal ultrasound was normal and had no nephrocalcinois. Urine screen for thiazide and loop diuretics was negative.

**Table 1:** Baseline her laboratory data after cisplatin treatment in 1986 and 2004

	June, 1986	January, 2004 ^▬^	Normal range
**Serum electrolyte levels**			
Sodium	133	140	136–145 mEq/l
Potassium	2.5	5.2^◇^	3.5–5.1 mEq/l
Chloride	89	105	98–107 mEq/l
Calcium	7.8	9.7	8.8–10.5 mg/dl
Magnesium	0.2	1.3	1.3–2.1 mg/dl
Creatinine	-	0.9	0.5–1.2 mg/dl
Serum bicarbonate level	35	26	20–30 mmol/l
**Serum pH**	**-**	7.5	7.30–7.50
**Urine Osmolality**		860	300–1,090 mosml/Kg H_2_0
**Urine electrolyte excretions**			
Sodium	-	234	40–220 mEq/day
Potassium	-	81.3	25–125 mEq/day
Chloride	-	221	110–250 mEq/day
Calcium	-	35	100–300 mg/day
Magnesium	-	57	18–130 mg/day
Urine Ca/Cr ratio	-	0.09	0.1–1.06 mmol/mmol
**Fractional excretion**			
Potassium	-	33.6%	
Magnesium	-	8.5%	
Calcium	-	0.6%	

^◇ ^Potassium values in 2004: 2.9, 3.4, 3.5, 4.6, 4.6, 5.0, 5.1 mEq/L

^▬ ^Medications: magnesium sulfate 4 grams IV weekly, and daily oral KCL 240 mEq, amiloride 5 mg, and aldactone 150 mg.

Her laboratory results are consistent with a diagnosis of Gitelman's syndrome, including hypomagnesemia, hypokalemia, hypocalciuria and metabolic alkalosis. She has been seen in our clinic for 18 months with persistent fatigue and occasional muscle aches. She now takes daily oral Mg oxide 3,500 mg, KCL 60 mEq, and aldactone 150 mg to maintain serum Mg^++ ^and K ^+^levels in the normal range.

## Discussion

Hypokalemic metabolic alkalosis with hypomagnesemia and hypocalciuria is a rare complication of cisplatin therapy. We reviewed the worldwide medical literature from 1966 to the present and identified 13 cases with these electrolyte abnormalities after cisplatin treatment [3-5]. The clinical and laboratory data of these cases and our case are summarized in 2. The median age of the patients is 21 yrs old. The cumulative dose of cisplatin ranged from 310 to 1,710 mg/m^2^. Except for our patient these cases have not developed symptoms. The mean serum potassium level is 3.4 mEq/l, and the mean serum magnesium is 1.4 mg/dl. Most patients have had chronic hypomagnesemia for at least for 2 years after treatment (range 1–20 years). The mean fractional urinary magnesium excretion is 6.9%, and the fractional urinary calcium excretion is 0.6%. Seventy percent of patients have a slightly decreased GFR after treatment. The biochemical abnormalities in these patients resemble Gitelman's syndrome (GS), an autosomal recessive renal tubular disorder first described by Gitelman in 1966 [2]. This syndrome is caused by inactivating mutations in the thiazide-sensitive Na-Cl co transporter (NCCT) gene in the distal convoluted tubule [2]. The defect in NCCT gene leads to NaCl wasting which stimulates the renin-angiotensin-aldosterone system, resulting in hypokalemic metabolic alkalosis [2]. The salt wasting due to NCCT mutation is high enough to lower blood pressure and protect patients from hypertension. However, the RAAS stimulation and the degree of volume contraction do not stimulate significant renal or systemic PGE2 production. Therefore, these patients have normal urinary PGE2 excretion [6]. Renal biopsy in these patients demonstrates juxtaglomerular hyperplasia, but this finding is non-specific, probably secondary to persistent sodium depletion and hyperreninnemia [6]. GS causes salt craving, musculoskeletal complaints, fatigue and dizziness. Progression to renal insufficiency is extremely rare in GS patients, and only one patient has developed end stage renal disease [2]. A few patients with GS have developed chondrocalcinosis probably from chronic hypomagnesemia [2].

**Table 2:** Laboratory data of Gitelman-like syndrome patients after cisplatin treatment.

**Ref.**	**Age/Sex**	**Type of Cancer**	**Cisplatin dose (mg/m**^2^**)**	**K**^+ ^**(mEq/l)**	**Mg**^++ ^**(mg/dl)**	**FE**_Mg _**(%)**	**FE**_Ca _**(%)**	**GFR (ml/m/1.73 m**^2^**)**	**Persist (yrs)**
1988 ^(3)^	22/M	Testicular	459–954^◆¤^	3.4–3.9^◆ψ^	1.4	8.9^■^	0.9^■^	88	2–6^◆¤^
1988 ^(3)^	36/M	Testicular	459–954^◆¤^	3.4–3.9^◆ψ^	1.2	8.9^■^	0.9^■^	100	2–6^◆¤^
1988 ^(3)^	39/M	Testicular	459–954^◆¤^	3.4–3.9^◆ψ^	1.1	8.9^■^	0.9^■^	117	2–6^◆¤^
1988 ^(3)^	25/M	Testicular	459–954^◆¤^	3.4–3.9^◆ψ^	1.4	8.9^■^	0.9^■^	69	2–6^◆¤^
1988 ^(3)^	27/M	Testicular	459–954^◆¤^	3.4–3.9^◆ψ^	1.1	8.9^■^	0.9^■^	105	2–6^◆¤^
1988 ^(3)^	24/M	Testicular	459–954^◆¤^	3.4–3.9^◆ψ^	1.6	8.9^■^	0.9^■^	85	2–6^◆¤^
1990 ^(4)^	8.5/M	Neuroblastoma	720	3.4	1.6	5.5	0.2	85	1.5
1990 ^(4)^	11/F	Neuroblastoma	1,710	3.7	1.8	3.4	0.1	102	3.5
1990 ^(4)^	8/M	Neuroblastoma	630	3.6	1.2	8.3	0.2	79	1
1991 ^(5)^	13* (4 patients)	Neuroblastoma Sarcomas	310–1,710^◆¤^	3.2 ^♣^	1.4^♣^	4.3^■^	0.42^■^	85^■^	1.5*
Our case	42/F	Ovarian	No data	3.4^◇^	1.3^◇^	8.5	0.6	80	20
Mean +/- SD	21.0 + 11.1		872 +280	3.4 +0.1	1.4 +0.2	6.9 +2.2	0.6 +0.3	89 +12	3.8 +4.5

◆ No individual patient data reported.

^¤ ^Use mean value to represent data for calculation of table mean.

^ψ ^Use lowest value to represent data for calculation of table mean.

■ Mean value for all patients from the same study.

♣ Calculated number from figure 1 in ref 5. ◇ Mean value of 1986–2004 data.

* Median value for 12 patients with cisplatin therapy in ref 5.

The mechanisms for hypocalciuria and hypomagnesemia in GS remain a matter of speculation [2]. Hypocalciuria may result from membrane hyperpolarization due to decreased Na^+ ^entrance into the cell, leading to activation of luminal voltage-gated Ca^++ ^channels and thus enhanced Ca^++^reabsorption. Another possibility is enhanced reabsorption of Ca^++^in proximal tubular segments secondary to hypovolemia [2]. Hypomagnesemia in GS is possibly explained by the observation that a complete block of NCCT by chronic thiazide treatment results in an increased rate of apoptosis in DCT cells in rats [2]. Therefore, patient with GS may have reduced DCT epithelial surface area, which limits magnesium reabsorption. Magnesium wasting in the proximal tubule and thick ascending limb should result in a fractional excretion greater than 10%. Metabolic alkalosis and hypokalemia may also have a role in magnesium excretion. Treatment of GS requires correction of the serum electrolyte abnormalities [2]. Most patients with GS require magnesium supplementation for life. Some also require potassium salts and/or anti-aldosterone medications to correct and maintain the serum potassium level [2]. Nonsteroidal anti-inflammatory drugs are usually not helpful [2]. However, recent studies demonstrate that COX-2 in the macula densa modulates renin secretion, and there is one case report which describes successful treatment of refractory hypokalemia with a COX-2 inhibitor (rofecoxib) [7].

The mechanism for cisplatin nephrotoxicity remains uncertain. Cisplatin could cause decreased protein synthesis, membrane peroxidation, mitochondrial dysfunction, and/or DNA injury and thereby cause tubular injury. However, a definite relationship between the DNA-binding and renal cell cytotoxicity has not been established yet [1]. The morphological studies in humans with cisplatin nephropathy demonstrate focal tubular necrosis predominantly in the distal convoluted tubule and the collecting ducts. The glomerulus has no obvious morphologic changes. Changes in the distal nephron include mitochondrial swelling, nuclear pallor, and occasional frank necrosis [1]. We suggest that cisplatin may cause DNA injury at the NCCT gene and DCT epithelial apoptosis which produces Gitelman-like syndrome. Although this syndrome occurs infrequently, cisplatin causes frequent renal dysfunction. Hypomagnesemia occurs in 40–100% of patients and a reduction in GFR occurs in 20–30% of patients. Solute diuresis and administration of cisplatin in divided doses or as a continuous infusion reduce nephrotoxicity [1]. Prompt repletion of magnesium deficits and administration of supplemental magnesium reduce the risk of adverse effects from hypomagnesemia. All the case reports we identified describe patients who probably received cisplatin in the 1980s. More attention to drug toxicity has possibly reduced the frequency of this syndrome.

## Conclusion

This case demonstrates that cisplatin can have permanent effects on tubular function and can cause significant morbidity. This syndrome should be considered in patients with unexplained electrolyte abnormalities and a history of remote therapy for malignancy.

## Competing interests

The author(s) declare that they have no competing interests.

## Authors' contributions

KP: Collected the data, drafted the manuscript; FA and SS: participated in the data collection and helped to draft the manuscript; KN: Conceived the idea, coordinated the data and helped in drafting and finalizing the manuscript. All authors have read and approved the manuscript.

## Pre-publication history

The pre-publication history for this paper can be accessed here:


